# Status quo of annotation of human disease variants

**DOI:** 10.1186/1471-2105-14-352

**Published:** 2013-12-04

**Authors:** Hanka Venselaar, Franscesca Camilli, Shima Gholizadeh, Marlou Snelleman, Han G Brunner, Gert Vriend

**Affiliations:** 1 CMBI, NCMLS, Radboud University Nijmegen Medical Centre, Nijmegen, PO Box 9101, Nijmegen, HB 6500, The Netherlands; 2 Department of Human Genetics, Radboud University Nijmegen Medical Centre, PO Box 9101, Nijmegen, HB 6500, The Netherlands

## Abstract

**Background:**

The ever on-going technical developments in Next Generation Sequencing have led to an increase in detected disease related mutations. Many bioinformatics approaches exist to analyse these variants, and of those the methods that use 3D structure information generally outperform those that do not use this information. 3D structure information today is available for about twenty percent of the human exome, and homology modelling can double that fraction. This percentage is rapidly increasing so that we can expect to analyse the majority of all human exome variants in the near future using protein structure information.

**Results:**

We collected a test dataset of well-described mutations in proteins for which 3D-structure information is available. This test dataset was used to analyse the possibilities and the limitations of methods based on sequence information alone, hybrid methods, machine learning based methods, and structure based methods.

**Conclusions:**

Our analysis shows that the use of structural features improves the classification of mutations. This study suggests strategies for future analyses of disease causing mutations, and it suggests which bioinformatics approaches should be developed to make progress in this field.

## Background

Recent years have seen an amazing improvement in Next Generation Sequencing (NGS) techniques. As a result, an increasing number of variations in the human genome, being either benign variants or disease causing mutations, have been found and have been stored in publicly accessible databases. dbSNP [1] is the primary database of genetic variation in the complete human genome whereas many Locus Specific Databases (LSDBs) [2] exist that are established for the collection, analysis, and distribution of disease related information. The Leiden Open-source Variation Database (LOVD)-system enables everyone to easily set up their own LSDB according to recommendations by the Human Genome Variation Society (HGVS) [3]. Currently (November 2012), LOVD hosts more than 476,000 variants, of which more than 110,000 are unique, in 5013 genes in 86 public LOVD installations. Other initiatives such as the 1,000 Genomes Project [4], the International HapMap project [5], PHENCODE [6], and the Human Variome Project [7] collect the information from these databases and combine it with information from other sources, such as the UCSC Genome Browser [8] or phenotypic information. Together, they aim to create a comprehensive overview of variation in the human genome. dbSNP contains over 52 million SNPs, (build 135, October 2011) and, as it has been estimated that SNPs occur about every 200-300 base-pairs [9], this number will continue to grow to ~15 million SNPs in any individual genome.

More than 60% of the ~6000 well understood genetic disorders that are related to DNA mutations in coding regions are caused by point mutations [9], so that it doesn’t come as a surprise that most bioinformatics efforts in the human genetics field have been directed towards them. Point mutations in proteins are the result of mutations in the DNA, and they are the main engine for evolution to arrive at novel functionalities. Most mutations are unfavorable for the species and thus weeded out over the eons. In a series of seminal articles Dayhoff and co-workers [10] determined the likelihood of each possible residue exchange and converted these data into a log odd matrix that became the basis of today’s popular programs such as Clustal [11] or BLAST [12]. Dayhoff reasoned that residue exchanges that are seen more often in a large set of aligned sequences are in general more likely to be observed as the result of evolution. In 1974, Grantham [13], reasoned that the likelihood that a mutation can be accepted in a protein is related to the similarity between the wild-type and the mutant residue type. He used three scores for important amino acid features (c, p, v for composition, polarity, and volume) to arrive at what is now commonly known as the Grantham matrix from which one can obtain the Grantham score for a mutation observed in a protein. The use of a scorings matrix has a series of limitations as was already hinted at in Grantham’s 1974 paper. One problem is that matrix values are an average of all possible mutation outcomes. A serine - > threonine mutation generally is not likely to be catastrophic, unless the serine is accidentally located in the active site of a serine protease. Many mutations that are highly acceptable at the surface of a protein can be devastating in its core. And finally, Grantham and Dayhoff determined their matrices based solely on information extracted from water soluble proteins, which makes them less applicable to mutations observed in membrane embedded (parts of) proteins. Asparagine, for example, is the least conserved residue in many Dayhoff-type matrices, but tends to be the most conserved amino acid in many transmembrane (parts of) proteins. The problems associated with the use of scorings matrices were first addressed by Ng and Henikoff who designed the SIFT software [14] that uses a multiple sequence alignment (MSA). SIFT is an improvement over the use of simple scorings matrices because the multiple sequence alignment implicitly contains information about the location of the studied mutation in the protein. The PANTHER software is also based on a MSA, but uses position-specific evolutionary conservation scores to predict mutation severity [15]. Similarly, the SVM-profile method in PhD-SNP uses MSA’s to obtain the frequencies of the wild-type and mutant residue in order to classify the variant [16]. Recently, methods that are species-specific have been developed, which shows that there is still room for improvement in the field of multiple sequence alignments [17].

To go back to the serine - > threonine mutation example, MSA-based programs like SIFT will see that the active site serine is fully conserved while many other serines in the molecule will be much more variable and thus less likely to be deleterious if mutated. The main reasoning behind the use of multiple sequence alignments is that if a residue is important in a protein, it is also likely to be important in the homologous proteins in many other species, and if something is important it remains conserved. The corollary is that if it is conserved, it must also be important and have a deleterious effect if mutated.

Obviously, there must be more information that can be extracted from multiple sequence alignments than just the degree of conservation and many groups have used machine learning techniques on data about known mutations and SNPs to obtain better methods to predict the severity of mutations. These methods indeed tend to work better than MSA based methods [16,18,19] but most machine learning methods have as disadvantage that the way in which they reach their conclusion remains unclear to the user.

SNPS&GO [20] combines support vector machine derived information from PANTHER, sequence and profile data, and GO terms. SNAP [19] predicts the functional effects of a mutation using biophysical characteristics of the mutated residue, evolutionary information obtained from PSI-BLAST and SIFT, Pfam profiles, predicted structural features, and annotations when available. MutPred [21] classifies mutations based on evolutionary information and transition frequencies obtained from SIFT and PSI-BLAST, Pfam profiles, and a series of structural descriptors that can be predicted from the sequence. SNPs3D [22,23] consists of two methods. The structure-based method analyzes a series of structural effects using the solved protein structure where possible. The sequence-based method uses a MSA generated by PSI-BLAST to build a sequence profile. Results are pre-calculated for known variants. For newly submitted variants only the sequence-method is used. nsSNPanalyzer [24] uses MSA’s from SIFT to obtain evolutionary information and combines this with structural information for the mutated residue and its environment as obtained from the structure in the ASTRAL database.

It is common practice in bioinformatics to compare methods when multiple methods exist that claim to solve the same question, and human mutation analyses are no exception. Thusberg et al. [18] evaluated nine different mutation analysis methods using a test-set of more than 40.000 pathogenic and neutral variants. Their results indicated that performance of the prediction methods can be affected by residue location, CATH secondary structure classification of the protein, and physicochemical properties of the wild-type and mutant residue, such as hydrophobicity and accessibility. They found that even though combining data from structure and MSA does not always improve performance, two of the best performing methods used a combination of structural, functional, and MSA-derived information for their predictions. However, there is no single method that could be rated as the best by all parameters that were used in this study.

Karchin [25] performed a test of 22 SNP annotation servers using a small set of mutations that were reported to be associated with disease in recently published articles. The results reveal that many of the servers nowadays disagree with each other, provide results that are difficult to understand, are biased towards nsSNPs, and do not always use the most up-to-date version of the data. Karchin concluded that a golden standard to train new methods is required and new methods should focus on users without bioinformatics background.

Ng and Henikoff [26] provide an overview of amino acid substitution (AAS) prediction methods available on the internet and their performances as reported in the original articles. The authors mention that the performance of a method strongly depends on the data sets in which the method was tested. Additionally, while comparing AAS methods one should also take the percentage of substitutions that can be predicted by the method, the coverage, into account. Methods that are purely based on 3D-structural features provide fewer predictions than sequence-based methods because for many proteins an experimentally solved structure is not available yet. Ng and Henikoff [26] propose a CASP-like experiment [27] to evaluate the performances of the available AAS prediction methods.

Mooney [28] recognised that the quality of the method will depend on the quality of the input data. Better characterized genes will result in better quality predictions. If only sequence data is available, SIFT is likely to provide the best predictions, but in case a structure is available PolyPhen will improve the analysis. According to Mooney, better training sets will be required to improve the prediction methods in the future.

In an extensive review Wang et al. compared 22 different methods, including a few that were not developed into freely-accessible webservers [29]. The authors suggest strategies to improve future methods and emphasize the fact that methods should be user-friendly and should provide an interpretation of the prediction results. The latter is what we focus on with HOPE.

A comparison of articles that compare methods reveals that most methods predict 70-85% of all mutations correctly, albeit that in most studies emphasis was on the analysis of true and false positives while true and false negatives did not in all studies get the attention they deserved. It is also clear that the outcome of any comparison depends critically on the selected test dataset.

Sunyaev et al. [30,31] reasoned that the more knowledge one has about a protein’s sequence, structure, and function, the more precise it should be possible to predict the effect of any mutation on that protein’s function. Their PolyPhen [31] web server was the first of a new generation of servers that can collect and combine information from many sources to draw a conclusion about the effect of a mutation. PolyPhen (and also the new PolyPhen-2 server [32]) uses structural features obtained from the 3D-structure (if available), sequence based features such as the location of active sites, transmembrane domains, and PSIC scores to classify a mutation as either benign, possibly damaging, or probably damaging [30,32]. Along this line, other methods have been developed that use predictions by other methods and combine them with their own selection of features. FunSAV, for example, uses machine-learning techniques to analyse mutations using a wide selection of features [33]. In a second step the prediction is combined with that of other well-known methods such as SIFT [14] and SNAP [19]. Similarly, SVM-3D is an extension of SNP&GO [20]. SVM-3D uses PANTHER [15] to predict conservation scores and combines them with structural features. The authors of both FunSAV and SVM-3D compared their method to other well-known tools that are either structure-based or sequence-based and show that using structural information improves the prediction of disease-related mutations. Wainreb et al. [34] argued that incorporating 3D-features is not always advantageous due to errors in the PDB, such as crystallization artifacts or incorrect oligomers. Their MuD-method elegantly solves this problem by allowing the user to interact with the program, for example to choose the correct multimer. A major disadvantage of the aforementioned methods is that they all require the availability of a solved protein structure.

We made the HOPE [35] software along similar lines, with as an extension that HOPE automatically builds homology models when no structure is available for the disease causing protein while the structure has been solved for any homolog. HOPE uses 3D-information when possible, but can also use sequence-based predictors in cases where no solved structure or modelling template is available. Obviously, when PolyPhen and HOPE are included in method comparisons then the choice of test dataset is even more critical than already mentioned in most of the aforementioned method comparison articles.

We wanted to know how well structure based mutation analysis methods perform in those cases where structure information is available. The rationale behind this question is that the number of human proteins for which 3D structure information is available, or can be obtained through homology modelling is growing rapidly. It therefore seems highly likely that the methods of choice in the near future will all be structure based. So by testing the strengths of today’s structure based methods we can get a glimpse of the options available to us soon, and by studying their weaknesses we can find out which research is needed to optimally analyse variants when -in the near future- 3D structure information will be available for the vast majority of the human exome.

## Results and discussion

We collected a test dataset of 61 proteins in which 181 mutations were observed that were experimentally proven to be causally related to a human disease phenotype. We extracted from the Expasy database [36] 46 (neutral) SNPs in these same proteins. We assume that a SNP, that is seen in more than 1% of the human population, is not causally related to a disease phenotype, so that we can call these 46 SNPs the negatives. We manually analysed all mutations, and the conclusions of this study are available at the HOPE results website [37]. We selected only mutations in proteins with a known 3D structure or homolog.

Table 1 shows the results of 11 mutation analysis programs on 227 mutations (181 damaging + 46 SNPs). It must be absolutely clear that this is not a comparison from a consumer report point of view because we only analyse mutations in proteins for which 3D information is available, and that clearly aids those methods that explicitly use this structure data. So, Table 1 is not a consumer report but merely a glimpse of what can be expected some years from now when structure information is likely to be available for most human proteins. A dataset of 61 proteins and 227 mutations obviously is not large enough to be called representative, but today we cannot do much better because on the one hand there aren’t that many studies available yet that include mutations in human proteins with a known structure, and on the other hand, checking 227 mutations manually is already an enormous task. Fortunately, the trends we see in Table 1 agree in general with the average of the trends we find in a series of articles that all were performed to obtain a statistically significant consumer report [18,25,28,38], so although our conclusions might be off in detail, they are most likely valid at a global level.

**Table 1:** Comparison of 12 different methods for mutation analysis

**Method**	**Pathogenic mutants**	**SNPs**	**Based on**
Grantham [13]	67,4	65,2	AA differences
PhD-SNP [16]	85,6	73,9	Conservation
Panther [15]	86,5	35,1	Conservation
SIFT [14]	87,8	64,4	Conservation
SNPs&GO [20]	72,5	77,8	Conservation, GO terms
SNAP [19]	83,4	56,5	Conservation, sequence predicted structure information
MutPred [21]	92,8	85,7	Conservation, sequence predicted structure information
nsSNPanalyzer [24]	74,5	67,6	Conservation, 3D structural features from homologs, AA properties
SNPs3D [22]	86,3	62,8	Conservation, structure information (pre-calculated from database)
PolyPhen-2 [32]	95,0	58,6	Conservation, 3D structural features from homologs, SwissProt annotations
HOPE [35]	96,1	76,1	Conservation, structural features from structure and homology models, SwissProt features, predictions, AA properties

Results of the mutation analysis on 181 pathogenic and 46 neutral variants by 11 different methods. Pathogenic mutants and SNPs are shown in percentage of correctly predicted cases. The numbers indicate percentages correctly (damaging for the pathogenic variants and benign for the SNPs) predicted mutations.

**Figure 1:**
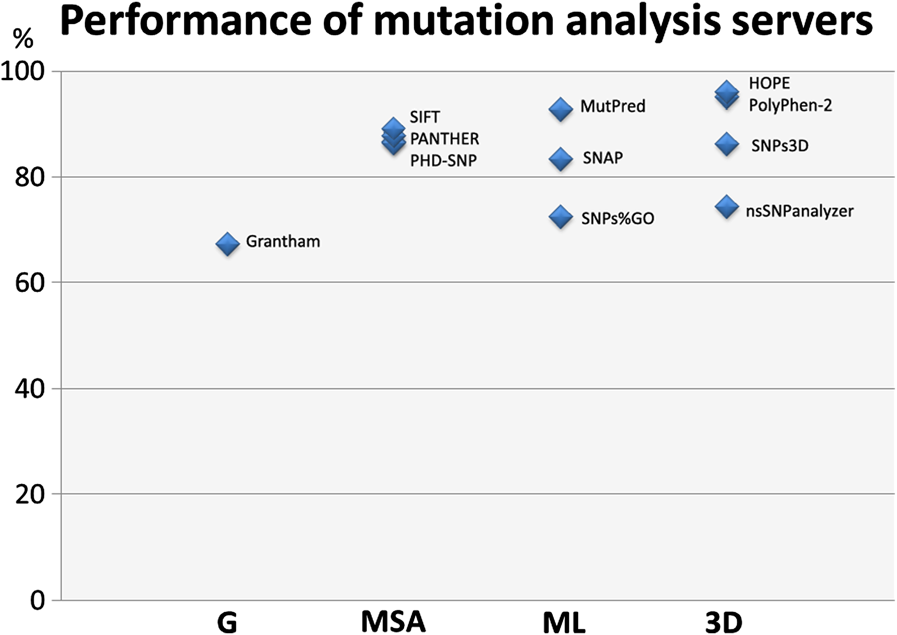
**Performance of the mutation analysis servers grouped by type.** Shown is the average percentage of the correctly predicted pathogenic mutants and neutral SNPs. The different servers are divided in groups based on their underlying methods. G = Grantham scores only, MSA = Multiple Sequence Alignment-centred methods (PhD-SNP, Panther, and SIFT), ML = Machine Learning based methods (SNPs&GO, SNAP, MutPred), 3D = structure based methods (nsSNPanalyzer, SNP3D, PolyPhen-2, and HOPE).

Table 1 and Figure 1 show a trend that using more information leads to better results. The very simple Grantham score performs poorest while the three methods that directly or indirectly use structure information perform best. Methods that augment MSAs with other information perform a bit better than SIFT that only uses the MSA. It is good to see that the two methods that make most use of the 3D-structures (PolyPhen-2 and HOPE) predict true positives all with a precision better than 90%. However, most methods tend to have many false positive predictions (with PANTHER even predicting 66,7% of the harmless SNPs as fatal). Again, the small size of the dataset makes that the methods cannot be compared in detail, but the trend is clear.

HOPE and PolyPhen are similar in how they obtain and analyse all data, but HOPE additionally writes a report about each mutation in layman’s terms. We compared these HOPE reports with the descriptions provided by the authors of the corresponding articles and with our own manual analyses. Among the mutations described in dataset 1 we found 12 cases in which the use of HOPE would have resulted in explanations of the mutation effects that are more detailed and/or more correct than those provided in the original articles. For sake of brevity we will describe just two, striking examples that will illustrate the value of a structural analysis. In both examples, a homology model was built using a template structure that was available to the authors at the time of submission of their article.

**Figure 2:**
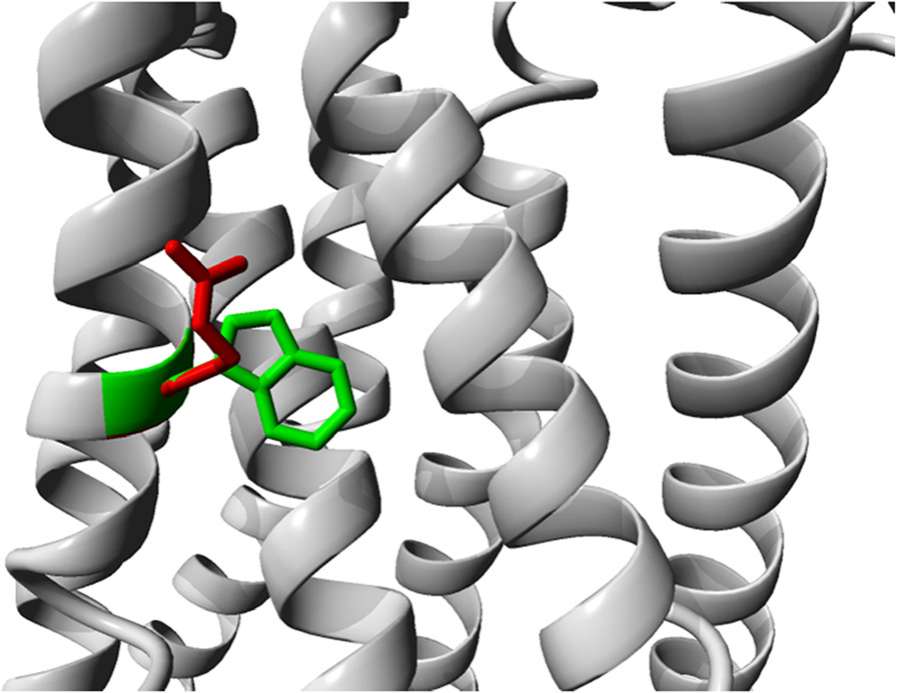
**Mutation W177R in opsin.** The opsin molecule is shown in grey, the side chain of wildtype tryptophan and mutant arginine are shown in green and red, respectively. The picture illustrates that the tryptophan residue is located at the surface where it can make hydrophobic interactions with the membrane.

W177R in opsin: In this case, a mutation of a big and hydrophobic residue into a charged residue at the surface of a transmembrane helix is very likely to affect the protein’s anchoring in the membrane. The authors, however, state that the mutation will “cause a major conformational change in the structure of the encoded protein” [39]. This is probably not correct as the side chain of the residue is not buried in the core of the protein, but instead is located at the surface where it interacts with the membrane lipids; arginine certainly will not make similarly favorable interactions (see Figure 2). The mutation will cause loss of hydrophobic interactions between tryptophan and lipids and will therefore affect the insertion or localization of the protein in the membrane. This is in agreement with the fact that the mutated protein was found to be retained in the ER. Most methods analyse this mutation correctly, but HOPE additionally explains that the mutation is located at the surface of a transmembrane domain where external interactions (in this case with lipids) are disturbed. PolyPhen-2 classifies this mutation as probably damaging.

**Figure 3:**
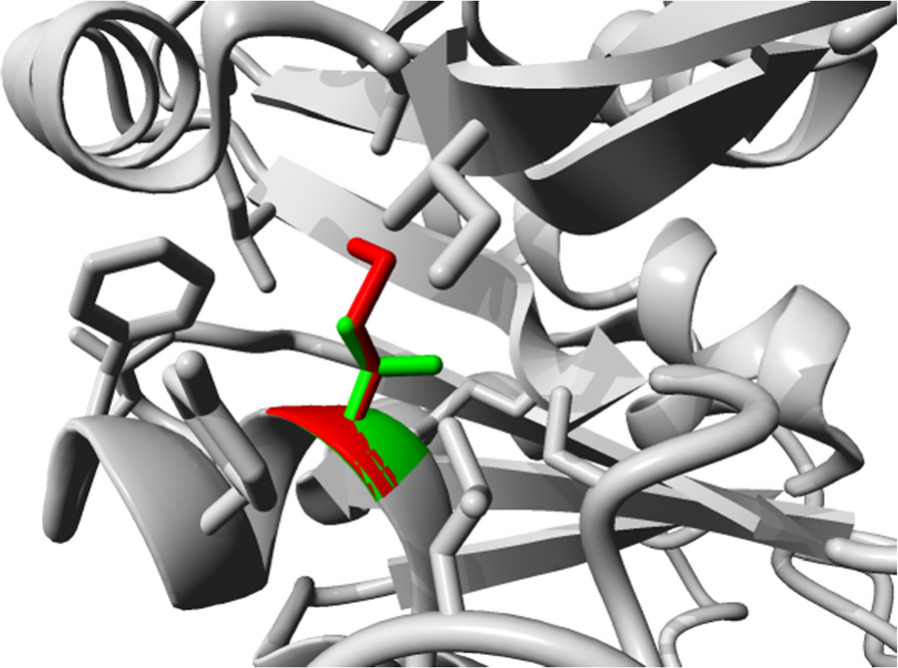
**Mutation V359 in SPTC2.** The SPTC2 molecule is shown in grey, the side chain of wildtype valine and mutant methionine are shown in green and red respectively. Other side chains of surrounding residues are also shown in grey and indicate that the residue is buried.

V359M in SPTC2: Valine is a hydrophobic residue that contributes to the stability of the protein’s core by making hydrophobic interactions. The homology model of the protein shows that the residue is buried and that a methionine will not fit at this position and thus will disturb the protein core (see Figure 3). The authors state that “The residue is a conserved amino acid residing in a conserved domains, possibly indicating functionally importance, located at the surface of the protein” [40]. HOPE’s use of the accessibility of the residue results in a hypothesis about the effect of the mutation. In this case, HOPE provides a highly plausible explanation for the structural origin of the observed effect. PolyPhen-2 also correctly predicts the mutation to be damaging.

Besides the two examples described above, we also found many cases in which the HOPE report provided extra information that underlined the author’s conclusions, simply by providing more details about the mutation. For example, Bem et al. [41] mention that the L24Q mutation in Rab18 will affect ligand binding. The HOPE report adds that this is caused by a change in size and hydrophobicity in the core of the protein which will affect ligand-binding residues in the vicinity of the mutation. In another case, Martinelli et al. [42] mention that that mutation Q376P will disturb the interaction at the RING-TKB interface. HOPE provides an extensive report that explains why the introduction of a proline in a helix and the loss of a hydrogen bond will affect the interface. These examples illustrate that the HOPE reports can provide more insight into the structural effects of mutations.

We found a series of possible points of improvement for all programs, including PolyPhen-2 and HOPE. For example, HOPE failed to identify the damaging effect for 3 mutations in the PRPS1 protein [43]. In all three cases, the original residue was not conserved and the mutant residue was also found at that particular position in the multiple sequence alignment. E43D and D65N both occur at the surface of the protein and were therefore classified by HOPE as neutral mutations. The HOPE program has an internal decision schedule that chooses the best structure from the Protein Data Bank for its analysis. When the PRPS1 sequence was submitted, HOPE identified a monomeric protein of PRPS1 as the best corresponding structure. Liu et al. [43] correctly used the multimeric biological assembly for their analysis and found that the mutated residues E43D and D65N are both located at the interface with the other subunits. Mutation of these residues will disturb this interaction and affect the function of the protein. The recent work by Wang et al. [44] beautifully illustrated the importance of protein-protein interactions for the analysis of disease-causing mutations. We added the use of biologically relevant protein complexes from the PISA-database in a new version of the HOPE-server.

Another example where HOPE fails is the third mutation in PRPS1. L129I, is predicted by HOPE as benign because leucine and isoleucine have the same properties and isoleucine was also observed at position 129 in the multiple sequence alignment. According to the authors this residue is located close to an allosteric binding site. The mutation might disturb this site and therefore affect the function of the protein. However, the exact location of this allosteric site is only described in literature, and not yet stored in any database that is accessible to HOPE. These examples illustrate the importance of correctly annotated and freely accessible data.

## Conclusions

It was stated recently by Lindblom and Robinson [45] that “the primary challenge in diagnostics in human genetics is likely to shift from the mere identification of human variation to the interpretation of these variants”. This is underlined by a recent editorial in Nature Genetics [46] that stresses the importance of “mechanistic investigation and further value” of disease-causing variants described in articles submitted for publication. In order to interpret the mechanistic effects of a disease-causing mutation one needs to collect from a wide variety of sources types of information such as conservation scores, location of coding regions and splice sites, the occurrence of other SNPs, functional sites in a protein, etc. Mutations can cause disorders in a variety of ways. For instance, a mutation that occurs in a regulatory motif could affect the recognition of that motif by the transcription-complex and thus affect transcription-regulation. A DNA mutation can also affect a mRNA splice-site which can lead to improperly functioning mRNA, change the localization signals in the pre-peptides, or even affect degradation of the protein which leads to aggregation of otherwise correctly functioning proteins. The majority of all known and characterized human inheritable disorders, however, are the result of a point mutation in the protein-coding region that leads to a protein that doesn’t function properly [47]. In order to fully understand the impact of a point mutation on the structure and function of a protein it is necessary to study the mutation in its spatial environment. Only by studying the 3D conformation of a protein in detail can we see whether it, for example, disturbs the structure of the active site, destabilizes a ligand-binding pocket, changes a dimerization-surface, or disturbs a disulphide bridge. In each case, the function of the protein will be affected in a different way and this knowledge can be beneficial for the development of drugs and therapies, or otherwise contribute to the aforementioned “mechanistic investigation and further value”. HOPE can meet the demand for more insight in mutations and their mechanisms as was proposed as a future research direction by Thusberg et al. [18] and the HOPE reports can form the starting point for new experiments that eventually lead to the design of new drugs/therapies, or even the repositioning of ‘orphan-drugs’ to cure the disease. The HOPE reports can be used by authors of articles that describe newly found mutations but also by the referees of those articles.

In this study we focus on proteins with a known structure, reasoning that the rapid increase of the PDB will soon make 3D structure information available for the majority of the human exome. However, disease related mutations have also been observed in natively unfolded (parts) of proteins. Some examples are the Aβ, α-synuclein and the prion protein that are major players in Alzheimer’s and Parkinson’s diseases and prion diseases, respectively. Like Aβ, α-synuclein is completely disordered, while prion proteins contain long disordered regions [48]. Three point mutations in α-synuclein (A30P [49], E49K [50], and A53T [51] are associated with the early onset of Parkinson’s disease and were shown to accelerate the α-synuclein aggregation (but not necessarily fibrillation) in vitro [52]. The ELM database [53] list a series of diseases related to mutations in so-called linear motifs. Most of these linear motifs are known located in NUPs. Examples are the Noonan [54], Usher [55], Liddle [56,57], and Golabi-Ito-Hall [58,59] syndrome. Surely, methods that base their variant analyses on protein structure information will need a special module to deal with mutations in natively unfolded (parts of) proteins.

The goal of this study was not to show which method works better, but rather to find out how much better the methods work that use 3D structure information. To prepare HOPE for the future, we still need to improve it in many ways. Like most methods, HOPE suffers from a too large number of false positive predictions. It therefore seems important to tune the software such that the ratio true positives plus negatives over false positives plus negatives gets optimised. This will undoubtedly reduce the now very high number of true positives but nevertheless increase the overall applicability of the method. During this study we realised the difficulty of separating loss of function from gain of function mutations. For example, mutations in the N-SH2 domain in PTPN11 were found to cause Noonan-syndrome. The N-SH2-domein interacts with the PTP-domain and thereby regulates the activity of PTPN11. Mutations in this area disturbed the interaction between the domains which results in an overall gain-of-function of the protein. In contrast to loss of function-mutations, the gain-of-function mutations do not have a detrimental effect on the protein structure and/or function. These mutation will remain difficult to classify automatically. Another difficulty is that most methods can only address one single point-mutation in one protein at a time while most complex diseases, such as cancer, can be caused by the combination of several common variants. Several methods have been developed for the disease-specific analysis of variations [60,61]. We expect that the automatic analysis of common variants related to complex disease will be a challenge for the future. With HOPE we focus on the 60% [9] of all human genetic disorders that are caused by one point mutation.

To improve HOPE further we will need to deal with all the aforementioned problems. We will also need to write special code for all kinds of (rare) events, like mutations at regulatory important cleavage sites, not yet annotated post-translational modification sites, or mutations at transient protein-protein interaction sites. The rewards of these efforts will be great because they will increase the percentage of correctly analyzed mutations. This in turn will even make HOPE a good tool for use in an emerging field like personalized medicine.

## Methods

### Dataset

A test dataset of 115 mutations was extracted from 34 recent articles published in the journals *Human Mutation, Nature Genetics* and the *American Journal of Human Genetics* that describe the effect of a disease causing mutation on the 3D-structure and/or function of the affected protein (dataset 1). As a negative control we used the 46 SNPs in the same set of proteins which have been annotated as non-damaging ‘polymorphisms’ at the UniProtKB/Swiss-Prot variant pages and which could be analysed using either the experimentally solved structure or a homology model. Additionally, we analysed 66 mutations that were studied manually in previous, in-house projects (dataset 2). Both datasets are available in Additional file 1: Table S1 and on the HOPE results website [37].

### Servers

All mutations were submitted to a series of servers. Many different servers exist and we realized it would simply be impossible to include them all in this study. Therefore, we made a selection based on a previous study by Thusberg et al [18], in which the authors compared the performance of 9 well-known mutation classifiers including: MutPred [21], nsSNPanalyzer [24], Panther [15], PhD-SNP [16], PolyPhen [30], PolyPhen-2 [32], SIFT [14], SNAP [19] and SNPs&GO [20]. The PolyPhen-server nowadays is offline, so we excluded it from the list. We added SNPs3D [22] an PMUT [62] to the list because they were used too in a series of consumer report articles [14,25]. However, results from PMUT yielded very low scores and the authors advised us not to use the software as they see no possibility to further maintain it. Therefore, we excluded this server from further analysis. Grantham scores were obtained using the table published in the original article [13]. The results of our extensive manual analyses of the structural and functional effects of the 181 disease causing mutants and 46 neutral SNPs are available at the HOPE results website [37].

### Automatic analysis and comparison

SIFT, PolyPhen-2, SNPs&GO, SNAP, and PhD-SNP produce simple to interpret answers that indicate whether a mutation is pathogenic or not. To interpret the results generated by SNPs3D, MutPred, Panther, and the Grantham scores, we used the threshold for pathogenicity as suggested by the authors in the corresponding articles. (Grantham: score > 62 = pathogenic, SNPs3D score > 0 = pathogenic, MutPred score > 0,5 = pathogenic, Panther score < -3 = pathogenic). HOPE was not designed to serve as a mutation classifier. To compare HOPE reports with the outcome of the other three methods the collected information was therefore translated to fatal/non-fatal. For instance, a report that mentions the loss of a salt-bridge at a conserved position clearly describes a damaging mutation which we therefore scored ‘pathogenic’ in this study. In contrast, a HOPE report that mentioned a non-conserved residue at the surface was scored as ‘benign’. At the HOPE results website we show the quantitative ‘recipe’ used for converting the HOPE reports into a binary pathogenicity score.

## Competing interests

The authors declare that they have no competing interests.

## Authors’ contributions

HV designed the study, drafted the manuscript and performed the statistical analysis. FC, SG and MS participated in the selection of the mutations and their analysis by the different methods. HB helped to draft the manuscript. GV supervised the study and helped to draft the manuscript. All authors read and approved the final manuscript.

## Supplementary Material

Additional file 1 **Table S1a and Table S1b.** Mutations used in this study. Click here for file
