# Supplementary material for: Status quo of annotation of human disease variants
Source: BMC Bioinformatics. 2013 Dec 4;14:352. doi: 10.1186/1471-2105-14-352 (PMC4234487; doi:10.1186/1471-2105-14-352)
Supplement: Additional file 1 — Table S1a and Table S1b. Mutations used in this study. [file 1471-2105-14-352-S1.docx]

**Sup. Table 1a – Mutations used in this study**

The mutations in this table were extracted from articles published in high-impact journals such as *Human Mutation, Nature Genetics* and *American Journal of Human Genetics*. The selected articles all described the structural effects of at least one point-mutation using either the 3D-structure or a homology model. We also searched the UniprotKB/Swissprot variant pages for variants in the same proteins that were annotated to be a polymorphism. These variants were used as a control-group.

| Protein accession code | Mutation | Damaging/Benign | Reference (PubMed-ID) |
| --- | --- | --- | --- |
| PGCA_HUMAN | V2303M | Damaging | E.L. Stattin (20137779) |
| SYYM_HUMAN | F52L | Damaging | L. G. Riley (20598274) |
|  | G191V | Polymorphism |  |
| CBL_HUMAN | Q367P | Damaging | S. Martinelli (20619386) |
|  | K382E | Damaging | S. Martinelli (20619386) |
|  | D390Y | Damaging | S. Martinelli (20619386) |
|  | R420Q | Damaging | S. Martinelli (20619386) |
|  | V904I | Polymorphism |  |
| PRPS1_HUMAN | D65N | Damaging | X. Liu (20021999 / A. de Brouwer (20380929) |
|  | A87T | Damaging | X. Liu (20021999 / A. de Brouwer (20380929) |
|  | I290T | Damaging | X. Liu (20021999 / A. de Brouwer (20380929) |
|  | G306R | Damaging | X. Liu (20021999 / A. de Brouwer (20380929) |
|  | D52H | Damaging | A. de Brouwer (20380929) |
|  | N114S | Damaging | A. de Brouwer (20380929) |
|  | L129I | Damaging | A. de Brouwer (20380929) |
|  | D183H | Damaging | A. de Brouwer (20380929) |
|  | A190V | Damaging | A. de Brouwer (20380929) |
|  | H192l,Q | Damaging | A. de Brouwer (20380929) |
|  | Q133P | Damaging | A. de Brouwer (20380929) |
|  | L152P | Damaging | A. de Brouwer (20380929) |
|  | E43D | Damaging | A. de Brouwer (20380929) |
|  | M115T | Damaging | A. de Brouwer (20380929) |
| OPSR_HUMAN | W177R | Damaging | C. Gardner (20579627) |
| PVRL4_HUMAN | T185M | Damaging | F. Brancati (20691405) |
|  | R284Q | Damaging | F. Brancati (20691405) |
|  | F53L | Polymorphism |  |
| DHB4_HUMAN | Y217C | Damaging | S. B. Pierce (20673864) |
|  | F90L | Polymorphism |  |
|  | R106H | Polymorphism |  |
|  | K140N | Polymorphism |  |
|  | T292S | Polymorphism |  |
|  | A427V | Polymorphism |  |
|  | A491T | Polymorphism |  |
|  | W511R | Polymorphism |  |
|  | I559V | Polymorphism |  |
|  | A606S | Polymorphism |  |
|  | T687I | Polymorphism |  |
|  | M728V | Polymorphism |  |
| SPTC2_HUMAN | G382V | Damaging | A. Rotthier (20920666) |
|  | V359M | Damaging | A. Rotthier (20920666) |
|  | I504F | Damaging | A. Rotthier (20920666) |
| SPCS_HUMAN | A239T | Damaging | O. Agamy (20920667) |
|  | Y334C | Damaging | O. Agamy (20920667) |
| SYK_HUMAN | L105H | Damaging | H.M. McLaughlin (20920668) |
|  | I274M | Damaging | H.M. McLaughlin (20920668) |
|  | G179A | Polymorphism |  |
| MASP1_HUMAN (isoform2) | G687R | Damaging | A. Sirmaci (21035106) |
|  | T21I | Polymorphism |  |
|  | V568A | Polymorphism |  |
|  | G679R | Polymorphism |  |
| MYLK_HUMAN | A1754T | Damaging | L. Wang (21055718) |
|  | S1759P | Damaging | L. Wang (21055718) |
|  | A1527V | Polymorphism |  |
| KLF1_HUMAN | E325K | Damaging | L. Arnaud (21055716 |
| PSB8_HUMAN | T75M | Damaging | A. K. Agarwal (21129723) |
|  | Q49K | Polymorphism |  |
|  | T74S | Polymorphism |  |
| CDN1B_HUMAN | P69L | Damaging | S. Molatore(20824794) |
| TRPV4_HUMAN | R269C,H | Damaging | G. Landoure(20037586) |
| RENI_HUMAN | D104N | Damaging | Annie Michaud(21036942) |
|  | S135Y | Damaging | Annie Michaud(21036942) |
|  | R33W | Polymorphism |  |
|  | Q160K | Polymorphism |  |
|  | G217R | Polymorphism |  |
| SPSY_HUMAN | G56S | Damaging | Z. Zhang(20556796) |
|  | V132G | Damaging | Z. Zhang(20556796) |
| PPA5_HUMAN | T89I | Damaging | T. Briggs(21217755) |
|  | G215R | Damaging | T. Briggs(21217755) |
|  | D241N | Damaging | T. Briggs(21217755) |
|  | M264K | Damaging | T. Briggs(21217755) |
|  | V148M | Polymorphism |  |
|  | V200M | Polymorphism |  |
|  | V221I | Polymorphism |  |
| CRGD_HUMAN | W43R | Damaging | B. Wang(21031598) |
|  | M102V | Polymorphism |  |
| I10R1_HUMAN | T84I | Damaging | E. O Glocker(19890111) |
|  | G141R | Damaging | E. O Glocker(19890111) |
|  | L61V | Polymorphism |  |
|  | V113I | Polymorphism |  |
|  | S159G | Polymorphism |  |
|  | R212Q | Polymorphism |  |
|  | I224V | Polymorphism |  |
|  | R351G | Polymorphism |  |
|  | S420L | Polymorphism |  |
| FXRD1_HUMAN | R352W | Damaging | E. Fassone(20858599) |
|  | V145I | Polymorphism |  |
|  | A343P | Polymorphism |  |
|  | H380R | Polymorphism |  |
| RASK_HUMAN (4B) | K5N | Damaging | L. Gremer(20949621) |
|  | V41I | Damaging | L. Gremer(20949621) |
|  | Q22E, R | Damaging | L. Gremer(20949621) |
|  | P43L, R | Damaging | L. Gremer(20949621) |
|  | T58I | Damaging | L. Gremer(20949621) |
|  | G60R | Damaging | L. Gremer(20949621) |
|  | D153V | Damaging | L. Gremer(20949621) |
|  | F156L | Damaging | L. Gremer(20949621) |
| DCTN1_HUMAN | G71A | Damaging | A. Weisbrich(17828277) |
| PGDH_HUMAN | A140P | Damaging | S. Uppal(18500342) |
|  | Y217C | Polymorphism |  |
| STXB1_HUMAN | V84D | Damaging | F. Deak(19255244) |
|  | C180Y | Damaging | F. Deak(19255244) |
|  | M443R | Damaging | F. Deak(19255244) |
|  | G544D | Damaging | F. Deak(19255244) |
| PH4H_HUMAN | F55L | Damaging | S.W. Gersting (18538294) |
|  | I65S | Damaging | S.W. Gersting (18538294) |
|  | H170E | Damaging | S.W. Gersting (18538294) |
|  | P275L | Damaging | S.W. Gersting (18538294) |
|  | A300S | Damaging | S.W. Gersting (18538294) |
|  | S310Y | Damaging | S.W. Gersting (18538294) |
|  | P314S | Damaging | S.W. Gersting (18538294) |
|  | R408W | Damaging | S.W. Gersting (18538294) |
|  | Y414C | Damaging | S.W. Gersting (18538294) |
|  | Y417H | Damaging | S.W. Gersting (18538294) |
|  | K274E | Polymorphism |  |
| DYR_HUMAN | L80F | Damaging | S. Banka (21310276) |
| FGF14_HUMAN | F145S | Damaging | J. van Swieten (12489043) |
| PTN11_HUMAN | D61Y, G, V, N | Damaging | M. Tartaglia (16358218) |
|  | A72T, V, S, G, I | Damaging | M. Tartaglia (16358218) |
|  | T73I | Damaging | M. Tartaglia (16358218) |
|  | E76K,Q,G,A,V,D | Damaging | M. Tartaglia (16358218) |
|  | L77V | Damaging | M. Tartaglia (16358218) |
|  | Y63C | Damaging | M. Tartaglia (16358218) |
|  | Y279C | Damaging | M. Tartaglia (16358218) |
|  | S502T, A, L | Damaging | M. Tartaglia (16358218) |
|  | G503R, L, E | Damaging | M. Tartaglia (16358218) |
|  | T468M | Damaging | M. Tartaglia (16358218) |
| MCFD2_HUMAN | D89N | Damaging | H. Elmahmoudi (21492322) |
|  | D81H | Damaging | H. Elmahmoudi (21492322 |
|  | V100D | Damaging | H. Elmahmoudi (21492322 |
| DHSA_HUMAN | R589W | Damaging | M.A. Pantaleo (21505157) |
|  | F33V | Polymorphism |  |
|  | D38V | Polymorphism |  |
|  | E240Q | Polymorphism |  |
|  | V657I | Polymorphism |  |
| DNAL1_HUMAN | N150S | Damaging | M. Mazor(21496787) |
| SYAM_HUMAN | R592W | Damaging | A.Gotz (21549344) |
|  | L155R | Damaging | A.Gotz (21549344) |
|  | I339V | Polymorphism |  |
|  | A484D | Polymorphism |  |
|  | M850V | Polymorphism |  |
| PRS56_HUMAN | R176G | Damaging | A.Gal (21397065) |
|  | W309S | Damaging | A.Gal (21397065) |
| RAB18_HUMAN | L24Q | Damaging | D. Bem (21473985) |
|  | N133S | Polymorphism | D. Bem (21473985) |

**Sup. Table 1b – Mutations used in this study**

A second set of mutations was obtained from proteins, studied in previous in-house projects. Most of our conclusions were published (see the column with references).

| Protein accession code | Mutation | Reference (PubMed-ID) |
| --- | --- | --- |
| HFE_HUMAN | H63D | D. Swinkels (18042412) |
|  | G93R | D. Swinkels (18042412) |
|  | I105T | D. Swinkels (18042412) |
|  | L183P | D. Swinkels (18042412) |
|  | C282Y | D. Swinkels (18042412) |
| EHMT1_HUMAN | C1042Y | T. Kleefstra (19264732 |
|  | R1166W | T. Kleefstra (19264732 |
| LRP5_HUMAN | E441K | K. Nikopoulos (20340138) |
|  | C1253F | K. Nikopoulos (20340138) |
| ERR2_HUMAN | L320P | R. Collin (18179891) |
|  | V342L | R. Collin (18179891) |
|  | L347P | R. Collin (18179891) |
| TOMT_HUMAN | R81Q | Z.Ahmed (18953341) |
|  | W105R | Z.Ahmed (18953341) |
|  | E110K | Z.Ahmed (18953341) |
| PO3F4_HUMAN | R329P | H. Lee (19671658) |
| TMPS6_HUMAN | C702F | - |
|  | R774C | - |
| NDUF3_HUMAN | G77R | A. Saada (19463981) |
|  | R122P | A. Saada (19463981) |
| SEC63_HUMAN | I120T | E. Waanders (20095989) |
|  | D168H | E. Waanders (20095989) |
|  | R267S | E. Waanders (20095989) |
|  | Q375P | E. Waanders (20095989) |
|  | W651G | E. Waanders (20095989) |
|  | D675E | E. Waanders (20095989) |
| KCNA1_HUMAN | N255D | J. van der Wijst (19903818) |
| TRPM6_HUMAN | G1955A | S. Thebault (18490453 ) |
| NDUV1_HUMAN | L53P | - |
|  | P122L | - |
|  | Y204C | - |
|  | C206G | - |
|  | A211V | - |
|  | R257Q | - |
|  | A341V | - |
|  | T423M | - |
| NDUS2_HUMAN | F84L | - |
|  | E104G | - |
|  | R228Q | - |
|  | P229Q | - |
|  | S413P | - |
|  | D446N | - |
| NDUS7_HUMAN | V122M | - |
| NDUS8_HUMAN | P79L | - |
|  | R94C | - |
|  | R102H | - |
| CABP4_HUMAN | R274C | K. Littink (19074807) |
| PCH15_HUMAN | R134G | Z. Ahmed (18719945) |
|  | D178G | Z. Ahmed (18719945) |
|  | G262D | Z. Ahmed (18719945) |
| SMAD3_HUMAN | T261I | I. van de Laar (21217753) |
|  | R287W | I. van de Laar (21217753) |
| TLR2_HUMAN | T411I | - |
|  | R579H | - |
|  | R753Q | - |
| CLC7A_HUMAN | I223S | B. Ferwerda (19864674) |
| ACAD9_HUMAN | E413K | J. Nouws (20816094) |
|  | R518H | J. Nouws (20816094) |
| EFGM_HUMAN | R250W | P. Smits (21119709) |
| COCH_HUMAN | F527C | - |
| TMPS3_HUMAN | A138E | N. Weegerink (21786053) |
|  | V199M | N. Weegerink (21786053) |
|  | A306T | N. Weegerink (21786053) |
|  | A426T | N. Weegerink (21786053) |
| IMPA3_HUMAN | D177N | L. Vissers (21549340) |
|  | T183P | L. Vissers (21549340) |
